# Prevalence and Risk Factors for Trachoma in Central and Southern Malawi

**DOI:** 10.1371/journal.pone.0009067

**Published:** 2010-02-05

**Authors:** Khumbo Kalua, Tobias Chirwa, Linda Kalilani, Sam Abbenyi, Mavuto Mukaka, Robin Bailey

**Affiliations:** 1 Department of Ophthalmology, College of Medicine, University of Malawi, Blantyre, Malawi; 2 Department of Mathematics and Statistics, Chancellor College, University of Malawi, Zomba, Malawi; 3 Research Support Centre, College of Medicine, University of Malawi, Blantyre, Malawi; 4 International Trachoma Initiative (ITI), New York, New York, United States of America; 5 Malawi Liverpool Wellcome Trust (MLWT), College of Medicine, Blantyre, Malawi; 6 London School of Hygiene and Tropical Medicine, London, United Kingdom; University of Cambridge, United Kingdom

## Abstract

**Background:**

Trachoma, one of the neglected tropical diseases is suspected to be endemic in Malawi. Objectives: To determine the prevalence of trachoma and associated risk factors in central and southern Malawi.

**Methodology/Principal Findings:**

A population based survey conducted in randomly selected clusters in Chikwawa district (population 438,895), southern Malawi and Mchinji district (population 456,558), central Malawi. Children aged 1–9 years and adults aged 15 and above were assessed for clinical signs of trachoma. In total, 1010 households in Chikwawa and 1016 households in Mchinji districts were enumerated within 108 clusters (54 clusters in each district). A total of 6,792 persons were examined for ocular signs of trachoma. The prevalence of trachomatous inflammation, follicular (TF) among children aged 1–9 years was 13.6% (CI 11.6–15.6) in Chikwawa and 21.7% (CI 19.5–23.9) in Mchinji districts respectively. The prevalence of trachoma trichiasis (TT) in women and men aged 15 years and above was 0.6% (CI 0.2–0.9) in Chikwawa and 0.3% (CI 0.04–0.6) in Mchinji respectively. The presence of a dirty face was significantly associated with trachoma follicular (TF) in both Chikwawa and Mchinji districts (P<0.001).

**Conclusion/Significance:**

Prevalence rates of trachoma follicles (TF) in Central and Southern Malawi exceeds the WHO guidelines for the intervention with mass antibiotic distribution (TF>10%), and warrants the trachoma **SAFE** control strategy to be undertaken in Chikwawa and Mchinji districts.

## Introduction

Trachoma is an infectious disease that is caused by serotypes A, B, Ba and C of the bacterium *Chlamydia trachomatis* [1], [2]. Repeated infections can eventually lead to eyelids thickening and developing scars resulting in the eyelashes turning inwards and rubbing on the cornea [3], causing abrasions and ulceration, eventually leading to visual loss and blindness. The World Health Organization (WHO) has categorized the clinical features of trachoma in five stages using the WHO simplified grading system [4], [5] as follows; Trachomatous inflammation follicular (TF), Trachomatous inflammation intense (TI), Trachoma Scarring (TS), Trachomatous trichiasis (TT) and Corneal opacity (CO).

Trachoma is still considered a leading cause of preventable blindness in sub-Saharan Africa especially in countries that have poor environmental sanitation, inadequate water supply and poor socio-economic status [6], [7]. Worldwide, despite improvements in trachoma control over the recent years, there are still about 8.2 million people with trachoma trichiasis and an estimated 40.6 million cases of active disease [6], [8]. Blinding trachoma remains a problem where living conditions facilitate continuous transmission of *Chlamydia trachomatis* among family members [9]. Indeed trachoma is among the many neglected tropical diseases (NTD's) that are associated with poverty and produce a disease burden almost as great as that associated with human immunodeficiency virus/AIDS, tuberculosis, or malaria [10] and may be treated simultaneously with other diseases.

The strategy recommended for control of trachoma is known as SAFE, an acronym standing for Surgery for Trichiasis, Antibiotic for active disease, Facial cleanliness promotion to reduce transmission from person to person by flies and Environmental improvement which reduces transmission of the infection. The WHO aims to achieve Global Elimination of Blinding Trachoma by the year 2020 (GET2020) through implementation of the SAFE strategy [11]. Countries like Malawi in Sub-Saharan Africa which had some districts in the Southern region with high prevalence rates of trachoma (TF >30%) over 20 years ago [12] but did not implement the SAFE strategy are still listed as trachoma endemic countries by WHO despite the lack of recent epidemiological data about the current overall prevalence of blinding trachoma. Trachoma is suspected to be endemic in Malawi even though there has been few prevalence population based studies, the last one done in Chikwawa in 1999 [13]. A lack of district level prevalence data exists in many other countries suspected of having endemic trachoma [14]. However some evidence suggests that prevalence of trachoma can be reduced over a period of time even if only parts of the SAFE strategy, rather than the whole strategy has been implemented [13]. There is therefore a need to conduct population based studies of trachoma in areas where the prevalence was assessed previously and some trachoma control activities implemented afterwards; this is necessary to document the changes over a period of time and give guidance as to whether there is still a need to implement the recommended SAFE strategy in an integrated manner. The integration of trachoma control strategies with other neglected tropical diseases (NTD's) may be beneficial in Sub-Saharan Africa [15].

We sought to determine the prevalence of trachoma in one previously highly endemic district (Chikwawa) and in one other district (Mchinji) in Malawi suspected of having trachoma but with no previous prevalence data.

## Methods

Ethical approval was obtained from the National Health Sciences Research Committee (NHSRC) based in Lilongwe, and the district health administrative offices (DHO) for Chikwawa and Mchinji Districts, in Malawi. Upon explanation of the purpose of the study, written informed consent was obtained from all subjects who participated in the study. Where the participant was a minor, written informed consent was obtained from the guardian.

The study districts were Chikwawa in the southern of Malawi, population 438,895 (National Statistical Office preliminary results, population and housing census, Zomba, Malawi 2008), known for high prevalence rates of trachoma [12], and Mchinji district in central region, population 456,558 and no trachoma survey done before.

**Figure 1:**
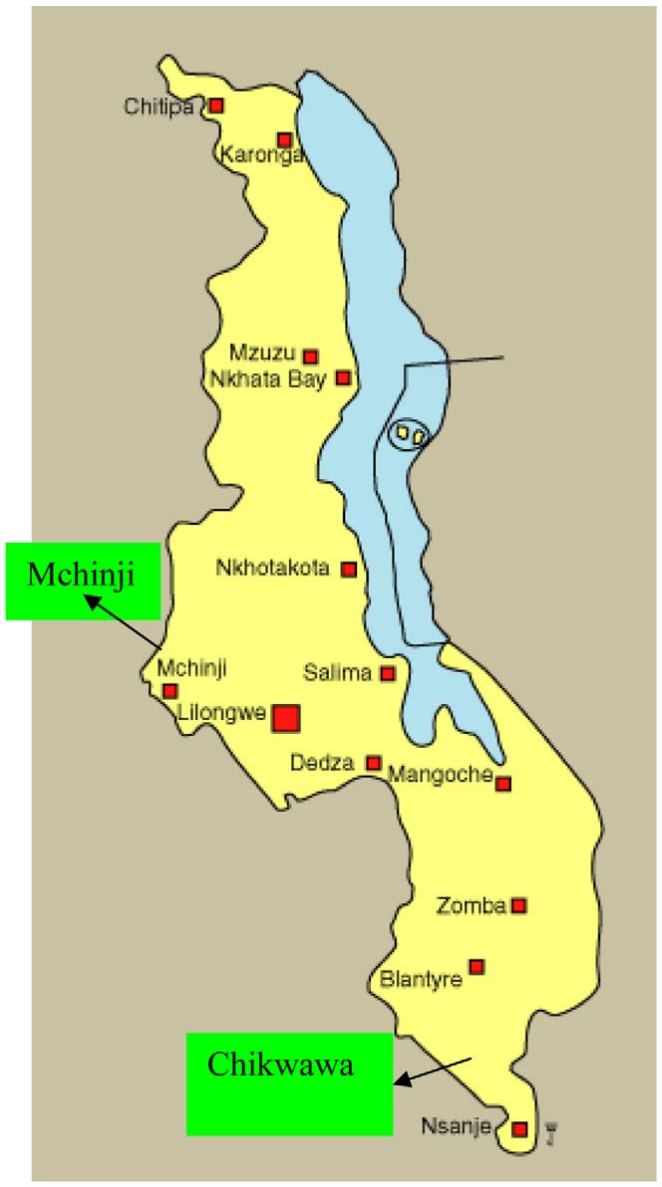
Map of Malawi showing survey sites.* *Mchinji in Central and Chikwawa in Southern Malawi.

Figure 1 shows map of Malawi and the districts where the study was done.

The 1988 trachoma study in Chikwawa district reported by Tielsch et al. found prevalence rates of TS to be 40% in those aged above 50 years while the prevalence of inflammatory trachoma (TF/TI) in children aged 1–2 years was nearly 50%. Hoechsmann et al. (14), in a study done in the same district 13 years later reported prevalence rates of trachoma follicles (TF) in those aged 1–6 years as being 13.9%.

Both Chikwawa district and Mchinji have a referral general hospital where an Ophthalmic clinical officer is based and regularly conducts eye clinics at the district hospital and also conducts outreach eye clinics at various health centres located within the community. The clinical officer is trained to do trichiasis surgeries within the district hospital using the WHO recommended bilamelar tarsal plate rotation. However coverage for trichiasis surgery is very low in both districts with less than 50 cases done per year, and this has been mainly attributed to lack of surgical equipment. Health promotion on primary eye care including hygiene and environmental control is regularly done by community health workers (Health Surveillance assistants) who have been trained and are fully based in the community. Trachomatous infections are currently treated with tetracycline eye ointment within the health centers and at the district hospitals. Both district hospitals have a strong environmental department which focuses on safe water and other hygienic programmes as part of their health promotion activities.

The study was a cross-sectional household based survey designed to obtain district level prevalence estimates for trachoma; TF and TI (collectively constituting active trachoma) in children aged 1–9 years; trichiasis (TT) in adult men and women aged 15 years and above, and to assess the predisposing risk factors. The study was conducted between July and September 2008.

### Sample Size

To calculate the sample size, the WHO recommended methodology for sample size calculation for trachoma surveys [16] was used. Using the following parameters: and an assumed prevalence of TF/TI of 20% for both districts, with the lowest expected rate being 16%, a significance level of 5%, [confidence of 95%], and a design effect of 4, the number of children aged 1–9 years and adults aged 15 years and above required for this study was 1728 children and 1414 adults per district and these were sampled concurrently. With an estimated average of 1.8 children per household in Malawi, and an assumed response rate of 90%, a total of 1054 households were needed in each district Accordingly 54 clusters (villages) per district were selected through probability proportionate to size sampling (PPS), using census data from the 1998 as the sampling frame, in which subsequently 20 households per cluster were sampled. A list was produced of the villages and their respective population sizes. A column was created with the cumulative population across the enumeration areas and the total population was divided by the number of clusters (54) required to derive the sampling interval. The first cluster was selected by multiplying the sampling interval with a random number between 0 and 1, the resulting number was traced in the cumulative population column, and the first cluster was taken from the corresponding village. Consecutive clusters were identified by adding the sampling interval to the previous number. The second stage of selecting 20 households within the cluster was done randomly, using a known list of all households within the cluster (collected by a community health worker) and a computer programme choosing 20 randomly selected households from the list.

### Data Collection

For data collection, 8 teams of paired ophthalmic clinical officers underwent a one week certification training session conducted by an expert on grading and trachoma surveys (RB) and observed by a member from the International Trachoma initiative (SA). Prior to the training, a baseline exercise involving a set of 50 digital eyelid photographs (derived from studies in Gambia, Tanzania and Ethiopia) were shown to the trainees with grade explanations and advice on grading. After training, a further set of 50 slides were projected and shown to the trainees, and they were asked to grade as taught during the training. Differences among trainees were examined and all queries were resolved. Trainees then went to the field for a pilot study, where they were observed examining and grading children and adults. After the field exercise, trainees were assessed using a final set of 50 digital photographs that were projected and graded independently by the trainer. A final Kappa score was obtained by comparing the grades of each trainee with the trainer (RB). For certification all trainee examiners were required to reach a kappa value of 0.61–0.8 (i.e. having fewer than 5 disagreements among the fifty slides).

Data was collected using 3 generic WHO standard forms for trachoma survey (village, household and ocular examination questionnaires). On arrival in the field, the first questionnaire was administered to village headman, the second questionnaire to the head of the household and the third questionnaires to all members of household. The village level questions focused on health facilities within the village, sources of water that the villages used and whether they had committees to oversee water issues in the villages, and schools that could be used as sources of health promotion in the area.

Each eye was examined for presence of trachoma using a 2.5 × magnifying loupe (Binomag plastic, Zabby's, India). Other data collected included the environmental factors such as availability of functional latrines, presence of solid waste or animal pens, distance to water source and hygienic condition of children faces (clean/unclean). A clean face was defined as absence of obvious ocular and nasal discharge.

Information on the questionnaires was double entered into an electronic Microsoft Access version 2003 database at a central location, cleaned and imported into Stata 10 (Stata Corp, Texas USA) software which was used for analysis. Survey logistic regression was used in order to adjust for clustering in the household. All tests were tested at 5% significance level. Firstly, univariate survey logistic regression analysis was performed to assess whether each of the following variables: clean face, water availability, privacy, sex, toilet, human faces, presence of kraal, means of waste disposal, radio and bicycle were associated with trachoma follicular (TF) in children aged 1–9 years. Secondly, multivariate logistic regression analysis was performed to assess which variables were independently associated with the outcome (TF).

## Results

A total of 1010 households in Chikwawa district and 1016 households in Mchinji district out of the planned 1054 households for each district were enumerated, giving an overall response rate of 96%. Overall a total of 6,792 persons (3,283 persons in Chikwawa and 3,509 persons in Mchinji) participated in ocular examination exercise. Among these 2,430 were children aged 1–9 years,1183 children aged 10–14 years and 3,179 were adults aged 15 and above.

Table 1 shows the baseline characteristics (sex and age range) of the study participants and Table 2 shows availability of household characteristics that were studied in the two districts. In regard to demographics of examined population in each cluster, overall there were 4.4 individuals per household (95% CI 4.31–4.57) in Chikwawa and 4.1 individuals per household (95% CI 3.95–4.20) in Mchinji respectively. As for children aged 1–9 years, there were 1.12 children per household in Chikwawa (95% CI 1.06–1.20) and 1.3 children per household in Mchinji (95% CI 1.21–1.34).

**Table 1:** Baseline characteristics of the study participants.

Variable	Chikwawa district	Mchinji district
	No. (%)	No. (%)
**Sex**		
Male	1599(48.7)	1688(48.1)
Female	1684(51.3)	1821(51.9)
**Age Group (years)**		
1–9	561(17)	622(18)
10–14	1587(52)	1592(45)
15+	1135(35)	1295(37)
**ALL**	**3283(100)**	**3509(100)**

**Table 2:** Availability of household characteristics in Chikwawa and Mchinji districts.

	Chikwawa	Mchinji
	No. (%)	No. (%)
**Type of Toilet**		
Pit Latrine	482(48.7)	629(62.2)
Flush Toilet	43(4.4)	2(0.2)
Other	6(0.6)	0 (0)
None	458(46.3)	380 (37.6)
**Faeces around 15 m of the house**		
Yes	71 (7.1)	6 (0.6)
No	924 (92.9)	1009 (99.4)
**Loose garbage around the house**		
Yes	446 (44.8)	166 (16.4)
No	549 (55.2)	849 (83.6)
**Water source**		
Tap water	114 (11.4)	98 (9.7)
Rain Water Harvest	1 (0.1)	1 (0.1)
Protected Well	17 (1.7)	104 (10.3)
Unprotected Well	98 (9.8)	421 (41.7)
Borehole	570 (56.9)	356 (35.3)
Dam	1 (0.1)	0
River/Stream	200 (19.9)	30 (3.0)
**Water all year round**		
Yes	734 (73.3)	762 (75.3)
No	267 (26.7)	250 (24.7)
**Distance to main water source**		
<15 minutes	666 (66.3)	810 (80.0)
16–30 minutes	248 (24.7)	190 (18.7)
31–60 minutes	77 (7.7)	13 (1.3)
>60 minutes	14 (1.4)	0

The prevalence rates of trachoma follicular (TF) and trachoma intense (TI) in children aged 1–9 years and trachoma scarring (TS), trachoma trichiasis (TT) and cornea opacity (CO) in adults aged 15 years and above in Chikwawa and Mchinji respectively are shown in Table 3 The prevalence of TF among children aged 1–9 years was found to be 13.6% (95% CI 11.6–15.6) in Chikwawa and 21.7% (95% CI 19.5–23.9) in Mchinji districts respectively. Taking into account the fact that children aged 1–9 years constituted 37% of the total population in the two districts; this translates into a total of 22,085 children from Chikwawa and 36,657 from Mchinji with trachoma follicles that need mass antibiotic treatment.

**Table 3:** Prevalence rates for Trachoma* in Chikwawa and Mchinji districts.

District	Children aged 1–9 years				Adults aged 15 and above					
	TF		TI		TS		TT		CO	
	%	CI**	%	CI	%	CI**	%	CI	%	CI
Chikwawa	13.6	11.6–15.6	2.0	1.1–2.8	6.0	3.5–7.5	0.6	0.2–0.9	0.6	0.1–1.1
Mchinji	21.7	19.5–23.9	3.4	2.2–4.4	4.8	3.5–5.1	0.3	0.04–0.6	0.2	0.8–4.7

* Trachoma Follicles (TF), Trachoma Intense (TI), in children aged 1–9 years; Trachoma scarring (TS), Trachoma Trichiasis (TT) and Cornea Opacity (CO) in adults aged 15 years and above.

** 95% confidence Intervals.

The prevalence of trachoma follicles (TF) among children aged 1–9 years by age group is shown in table 4:

**Table 4:** Prevalence of Trachoma follicles (TF) by age in Chikwawa and Mchinji districts.

	Chikwawa		Mchinji	
Age(years)	Examined	With TF (%)	Examined	With TF (%)
1	114	10(8.8)	145	22(15.2)
2	134	23(17.2)	191	51(26.7)
3	156	33(21.2)	171	44(25.7)
4	162	22(13.6)	172	46(26.7)
5	148	19(12.8)	163	42(25.8)
6	109	19(17.4)	123	20(16.3)
7	133	11(8.2)	109	24(22.0)
8	105	11(10.5)	145	25(17.2)
9	74	6(8.1)	76	7(9.2)
Total	1135	154(13.6)	1295	281(21.7)

Table 5 shows the prevalence of trachoma trichiasis (TT) by sex among adults aged 15 years and above in Chikwawa and Mchinji districts respectively. The prevalence of trachoma trichiasis (TT) was 0.6 (CI 0.2–0.9) in Chikwawa and 0.3 (CI 0.04–0.6) in Mchinji district respectively. There was no significant difference in prevalence rates for trichiasis between men and women in both districts. Taking into consideration that adults aged 15 and above contributed to 45% of total population in both districts, prevalence rates of trichiasis obtained translate to a total of 1580 and 616 cases of people with trichiasis needing surgery in Chikwawa and Mchinji districts respectively.

**Table 5:** Prevalence of Trachoma Trichiasis (TT) by sex in Chikwawa and Mchinji districts.

Chikwawa	No examined	No with TT	% (*CI)	P value
Males	613	1	0.2(−0.2,0.6)	0.17
Females	979	8	0.8(0.3,1.2)	
Total	1592	9	0.6(0.2,0.9)	

Mchinji	No examined	No with TT	% (*CI)	P value
Males	631	2	0.3(−0.1,0.7)	1.00
Females	1020	3	0.3(−0.04,0.6)	
Total	1651	5	0.3(0.04,0.6)	

* 95% confidence Intervals.

Table 6 shows risk factors for trachoma follicular (TF) obtained through univariate and multivariate logistical regression after accounting for clustering in Chikwawa and Mchinji districts respectively. Risk factors for trachoma follicular (TF) obtained through univariate logistical regression after accounting for clustering in households in Mchinji were dirty face (OR 3.66, CI 2.20–6.09, P<0.001), presence of water throughout the year (OR 0.36,CI 0.17–0.75, P = 0.006), and no privacy to toilet (OR 3.29, CI 1.52–7.13,P = 0.003), while on multivariate analysis only dirty face (OR 6.54, CI 1.20–35.63, P = 0.046) and no privacy to toilet (OR 18.47, CI 2.1–160.1, P = 0.009) were significant. In Chikwawa, significant risk factors obtained on univariate logistical regression were dirty face (OR 1.94, CI 1.45–2.85, P<0.001), age (OR −0.06, CI −0.005,−0.12, P = 0.03) and being female (OR 0.65, CI 0.49–0.87, P = 0.004) but nothing was significant on multivariate regression.

**Table 6:** Risk factors for Trachoma follicular (TF) obtained through univariate and multivariate logistical regression.

			Univariate	analysis			Multivariate	analysis
**Chikwawa**								
Variable	No	OR	CI*	P-value	No	OR	CI*	P-value
Age(1–9)	1122	−0.06	−0.12,−0.005	0.03	246	0.91	0.80,4.4	0.167
Sex (Female)	1122	0.65	0.49, 0.87	0.004		0.56	0.25,1.21	0.139
Presence of dirty face	1096	1.94	1.45, 2.85	<0.001		1.72	0.82,3.61	0.148
No radio in household	1043	1.31	0.91, 1.89	0.144				
No bicycle in household	1043	0.49	0.57, 1.25	0.392				
**Surrounding area**								
Absence of toilet	1029	0.83	0.57, 1.21	0.328				
Toilet with no privacy	538	1.08	0.58, 2.02	0.801				
Presence of feces	1040	1.69	0.80, 3.58	0.172				
Presence of Kraal	496	0.59	0.35, 1.00	0.052				
Presence of garbage	1028	0.54	0.28, 1.04	0.067				
**Source of water**	1042							
Unprotected well		1.38	0.68, 2.82	0.368				
Borehole		0.81	0.45, 1.45	0.476				
River		0.56	0.28, 1.10	0.091				
**Mchinji**	459							
Age (years)		−0.05	−0.14, 0.04	0.25				
Sex (Female)		0.76	0.49, 1.19	0.49				
Presence of dirty face		3.66	2.20, 6.09	<0.001	74	6.54	1.20,35.63	0.046
No radio		0.89	0.54, 1.49	0.67				
No bicycle		0.89	0.54, 1.47	0.65				
**Surrounding area**								
Absence of toilet	379	0.98	0.58, 1.65	0.94				
Toilet with no privacy	228	3.29	1.52, 7.13	0.003	74	18.47	2.1–160.1	0.009
Presence of Kraal	166	0.81	0.39, 1.70	0.58				
**Source of water**	386							
Unprotected well		0.63	0.27, 1.47	0.29				
Borehole		0.78	0.33, 1.86	0.58				
Water lasts all year		0.36	0.17, 0.75	0.006	74	0.74	0.28,1.96	0.55

* 95% confidence Interval.

In regard to sources of water, after accounting for clustering in the households, the main source of water for Mchinji was unprotected well (42%) and for Chikwawa it was borehole (57%) (Table 2). Health facilities (hospitals, health centres, and health posts) and schools were evenly distributed in Mchinji and Chikwawa districts with each cluster having access to services within a distance of 5–10 kilometres.

## Discussion

This study was conducted to determine the prevalence of trachoma infections (TF/TI) among children aged 1–9 years, the prevalence of trachoma scaring (TS),trachoma trichiasis (TT) and cornea opacity (CO) among adult men and women aged 15 years and above and association with the risk factors for trachoma in Chikwawa and Mchinji districts. To our knowledge this is one of the largest and most recent trachoma population based Ophthalmological survey undertaken in Malawi (total persons examined was 6,792), the first large population based study of trachoma done in Malawi was conducted in Chikwawa more than 20 years ago [12]. However several other studies have reported the prevalence on trachoma in some areas of Malawi even though some were not specifically designed for trachoma [17], [18]. The results of this study show that trachoma follicular infections (TF) is still a disease of public health importance despite a marked reduction in prevalence in Chikwawa over a 20 year period (from 50% to 13.6%), and these results are in agreement with a relatively recent study by Hoechsmann et al.(14) who reported a prevalence of active trachoma in Chikwawa in children aged 1–6 years as 13.9%. One limitation of comparing the present study with the earlier ones is the difference is the age range of children examined, the current study using the WHO age range of children from 1–9 years versus 1–6 years in earlier studies.

The prevalence of trachoma follicular (TF) found among children aged 1–9 years in both districts exceeds the WHO guidelines for the intervention with mass antibiotic distribution (TF>10%), and warrants the SAFE strategy to be undertaken to address the issue of trachoma in Chikwawa and Mchinji districts.

The prevalence of trichiasis (TT) observed in adults aged 15 years and above in Chikwawa (0.6%) and Mchinji (0.3%) has overlapping confidence intervals indicating that the rates were not significantly different in the two districts. However the values are higher than WHO cut off point of threshold for elimination of trichiasis (TT) as a public health problem (0.1%) and indicate that surgical intervention for trichiasis should be implemented in the two districts.

Indeed the obtained prevalence rates of trichiasis translate to large numbers of people needing surgical intervention for trichiasis in Chikwawa and Mchinji districts (1580 and 616 respectively), and some of these may end up developing cornea opacity (CO) and becoming blind if not surgically corrected. It is important to note that not all cases of trachomatous trichiasis progress to cornea opacity, and that some of the cornea opacities seen in the community are not caused by trachoma; but may be caused by other cornea infections and concurrent use of traditional herbal medicines for treatment. However it is most likely that cases of cornea opacities seen in the two districts are as a result of complications of trachoma trichiasis, as the uptake and surgical coverage of trichiasis surgery in the two districts is very low. Findings from talking to ophthalmic staff and other health workers from the two hospitals in the districts indicated that only a few trichiasis surgeries were being done, and this was mostly due to lack of trichiasis surgical equipment in the hospitals.

In regards to risk factors, although lack of water and long distance to water source is associated with active trachoma (TF/TI) [9], [19], [20] it is apparent from findings of this study that long distance to a water source was not a major factor in the transmission of trachoma in both districts as more than 90% of population had access to a water source of a distance of less than 30 minutes of walking. This finding is in contrast to Hoechsmann et.al (13) ] who found that trachoma follicular was associated with a longer distance to the primary water source in the Chikwawa district. It should be noted however that since the Hoechsmann study, many boreholes were drilled in Chikwawa district resulting in most residents accessing water within a walking distance of 30 minutes. The hypothesis that reduction in trachoma follicular (TF) was related to environmental improvement in absence of antibiotic treatment is indeed supported by the readily availability of water and not the antibiotic. Currently both districts have access to only limited amounts of tetracycline eye ointment as an available drug and only use it for treating severe eye infections.

One limitation in interpreting the association of risk factors to active trachoma is that some of the factors studied (availability of water sources, pit latrines, garbage disposal and animal pens) were collected at household and village level, and may not be applicable to individual children. We do not still fully understand why particular individual children within the same environment and sometimes within same households are more prone to have active trachoma than others

The relatively lower rates of trachomatous infection found in Chikwawa compared with Mchinji coupled with findings that there was readily available water in Chikwawa than Mchinji may explain the decline of prevalence of trachoma in Chikwawa in absence of a trachoma control programme using the SAFE strategy. Chikwawa district has for many years been popularly known as the “home of trachoma in Malawi”, with previous prevalence estimates of TF nearly 50% in children aged 1–2 years [12]. Results from the present study suggests that the prevalence of TF has markedly declined despite not having trachoma SAFE strategy programmes in the area and this observation has also been noted by Hoechsmann and colleagues [13] who attributed the reduction of TF in absence of antibiotic mass distribution to be due to presence of water and hygiene programmes which were initiated in their study area.

There was a strong correlation on logistical regression between prevalence of trachoma follicles (TF) and presence of dirty face in children in both Chikwawa and Mchinji (P<0.001), and no privacy to toilet in Mchinji but there was no correlation between presence of TF and waste disposal, animal pens and presence or absence of toilet. However it has been noted that garbage disposal in the surrounding area and presence of cattle feces may be associated with presence of *Musca sorbens*, the flies that are responsible for the transmission of trachoma [21].

In summary, the prevalence of trachoma found in Chikwawa and Mchinji districts confirms that trachoma is still a disease of public health importance in the two districts and calls for implementation of the full SAFE strategy. It is important to note that certain activities of the SAFE strategy (antibiotic distribution) may be integrated with control of other diseases and some studies have reported success [22]. The Ministry of Health in Malawi is controlling Onchocerciasis, Schistomiasis and lymphatic filariasis (LF) through community-directed treatment with multi-drug therapy and has systems in place that would possibly allow extension of integrated neglected tropical diseases (NTDs) control to include the antibiotic component of the trachoma control. However this should only be done when plans for implementing other components of the SAFE strategy have been fully put in place.

There are many similarities in the distribution and disease pattern of trachoma observed in the two districts. Chikwawa is located in the Southern region (south west zone) and Mchinji is located in the Central Region (central east zone) of Malawi and it may be possible that the prevalence of trachoma may be similar in other districts located within the same regions. However findings from this study cannot be easily generalized to the rest of other districts in Malawi as the two districts were selected only from the two out of the three regions in Malawi. Therefore carefully designed national surveys to cover randomly selected districts suspected of having trachoma in all the three regions should be planned and conducted to generate prevalence data at national level.
